# Synovial fluid but not plasma interleukin-8 is associated with clinical severity and inflammatory markers in knee osteoarthritis women with joint effusion

**DOI:** 10.1038/s41598-021-84582-2

**Published:** 2021-03-04

**Authors:** María García-Manrique, Joan Calvet, Cristóbal Orellana, Antoni Berenguer-Llergo, Silvia Garcia-Cirera, Maria Llop, Néstor Albiñana-Giménez, Carlos Galisteo-Lencastre, Jordi Gratacós

**Affiliations:** 1 Rheumatology Department, Parc Taulí University Hospital, I3PT Research Institute (UAB), c/Parc Taulí s/n, edifici VII Centenari, 08208 Sabadell, Spain; 2 Departament de Medicina, Universitat Autónoma de Barcelona (UAB), 08003 Barcelona, Spain; 3 Biostatistics and Bioinformatics Unit, Institute for Research in Biomedicine Barcelona (IRB Barcelona), 08028 Barcelona, Spain; 4 I3PT Research Institute (UAB), 08208 Sabadell, Spain

**Keywords:** Musculoskeletal system, Rheumatic diseases

## Abstract

Several cytokines and adipokines are related to clinical severity and progression in knee osteoarthritis. The aim of this study was to evaluate the associations of IL-8 with clinical severity and with local and systemic adipokines and cytokines. This is a Cross-sectional study including 115 women with symptomatic primary knee osteoarthritis with ultrasound-confirmed joint effusion. Age, symptoms duration and body mass index were collected. Radiographic severity was evaluated according to Kellgren–Lawrence. Pain and disability were assessed by Lequesne and Knee injury and Osteoarthritis Outcome Score pain, symptoms and function scales. Three inflammatory markers and five adipokines were measured by ELISA in serum and synovial fluid. Partial correlation coefficient (PCC) and corresponding 95% confidence interval were used to evaluate association. Synovial fluid IL-8 was significantly associated with clinical severity scales. After controlling for potential confounders, associations measured by a Partial Correlation Coefficient (PCC) remained essentially unaltered for Lequesne (PCC = 0.237), KOOS pain (PCC = − 0.201) and KOOS symptoms (PCC = − 0.209), KOOS function (PCC = − 0.185), although the later did not reach statistical significance. Also in synovial fluid samples, associations were found between IL-8 and TNF (PCC = 0.334), IL6 (PCC = 0.461), osteopontin (PCC = 0.575), visfatin (PCC = 0.194) and resistin (PCC = 0.182), although significance was not achieved for the later after statistical control for confounders. None of these associations were detected in serum. In conclusion, IL-8 was associated with clinical severity, inflammatory markers and adipokines in synovial fluid, but not in blood. Although the reported associations are weak to moderate in magnitude, these findings reinforce the notion that local and not systemic inflammation is more relevant to clinical severity in knee OA women with joint effusion.

## Introduction

Osteoarthritis (OA) is the most prevalent joint disease and the leading cause of pain and disability in adults^1^. The factors associated with the presence, severity and progression of OA are not well known. Among others, inflammatory factors have been related to OA over the past few decades, but their effects vary widely depending on the different studies^2^. Several previous studies have related some cytokines such as TNF-alpha or IL-6 to OA severity, both in synovial fluid and in serum^3^. However, differences according to disease duration and radiological stage in those studies hinders drawing definite conclusions. Adipokines such as leptin, adiponectin, resistin, visfatin and osteopontin in synovial fluid and plasma have been linked to clinical severity and to knee OA (KOA) progression^4–6^. Again, differences occur in the series published concerning disease duration, radiographic stage and the presence of synovial effusion that make hard to extrapolate conclusions.

Interleukin-8 (IL-8), also known as CXCL-8, is an inflammatory chemokine present under pathological conditions. It is produced by human OA chondrocytes and is considered to play an important role in OA pathophysiology as it mediates the release of matrix metalloproteinase-13 and has been related to inflammatory changes in the synovium. In a previous study using cartilage culture, IL-8 has been shown to induce hypertrophy and differentiation of chondrocytes in OA^7, 8^. It has also been reported that IL-8 synovial fluid levels are increased in OA patients compared to a control group of younger individuals with anterior cruciate ligament injury, observing that elevated IL-8 plasma is linked to MMP-3 activation^9^. However, to our knowledge no clinical associations between IL-8 and clinical severity has been evaluated in knee OA.

The aim of this study was to evaluate the associations of IL-8 in synovial fluid and plasma with clinical severity in KOA patients with joint effusion. As patients with joint effusion may represent a subset with a higher inflammatory component, we have also studied relevant cytokines and adipokines known to play a role in clinical severity and local inflammation in KOA.

## Results

### Subject characteristics

Included women with inflammatory KOA had a median age of 68.8 ± 11.1 years old with KOA symptoms duration of approximately 4 years. The median Body Mass Index (BMI) was within the obesity range (30.5 kg/m^2^). The Kellgren–Lawrence (KL) grades 2 and 3 were predominant in this cohort (41.7% each) while only 3.5% had KL grade 4. Moderate to high disease activity resulted from the different clinical questionnaires administered to the patients. Strong technical effects associated with round of measurement were identified in the quantification of the inflammatory factors and adipokines, and a statistical correction was carried out to enable comparison between samples (See “Assessments” section). Nevertheless, no cut-off points or comparison between plasma and synovial fluid determinations could be reliably established. The corrected values are shown in Table 1.

**Table 1:** Demographic variables, radiographic severity, clinical questionnaires, inflammatory markers and adipokines in synovial fluid and blood.

Variables	Category	Median (IQR)/N (%)
Demographics	Age	68.80 (11.1)
	KOA symptoms duration (months)	50.00 (73.00)
	BMI (kg/m^2^)	30.5 (6.35)
Radiographic severity	**KL**	
	1	15 (13.1%)
	2	48 (41.7%)
	3	48 (41.7%)
	4	4 (3.5%)
Clinical questionnaires^a^	Lequesne	14.00 (5.00)
	KOOS pain	42.00 (17.00)
	KOOS symptoms	44.00 (18.50)
	KOOS function	46.00 (19.00)
Inflammatory markers SF^b^	IL-8 SF pg/mL	10.96 (14.00)
	TNF-α SF pg/mL	10.19 (7.97)
	IL-6 SF pg/mL	105.99 (302.64)
Blood inflammatory markers^b^	IL-8 blood pg/mL	3.13 (2.46)
	TNF-α blood pg/mL	5.64 (1.95)
	IL-6 blood pg/mL	2.72 (3.31)
Adipokines SF^b^	Leptina SF pg/mL	42,079.42 (29,565.99)
	Adiponectin SF ng/mL	1734.76 (1352.53)
	Resistin SF pg/mL	2225.65 (2205.79)
	Visfatin SF ng/mL	1.53 (1.18)
	Osteopontin SF ng/mL	57.66 (83.19)
Blood adipokines^b^	Leptin blood pg/mL	37,948.02 (28,243.77)
	Adiponectin blood ng/mL	14,294.05 (9990.22)
	Resistin blood pg/mL	2068.29 (1173.56)
	Visfatin blood ng/mL	3.78 (1.37)
	Osteopontin blood ng/mL	10.97 (7.02)

Medians and interquartilic ranges (IQR) were used to describe continuous variables; categorical data were summarized using absolute frequencies (N) and percentages (%); *KOA* knee osteoarthritis, *KL* Kellgren–Lawrence scale, *BMI* Body Mass Index, *KOOS* Knee injury and Osteoarthritis Outcome Score, *SF* synovial fluid, *IL-8* interleukin-8, *IL-6* interleukin-6, *TNF-α* Tumor Necrosis Factor-alpha.

^a^Clinical questionnaires range values and interpretation: Lequesne (range from 0 to 24; where 0 is non and 24 maximum degrees of pain or incapacity) KOOS pain, symptoms, and function (range from 0 to 100; where 0 is maximum and 100 minimum of each condition evaluated).

^b^Levels of inflammatory markers and adipokines in synovial fluid and blood were corrected a-priori by measure round.

### Association between IL8 and clinical severity

Synovial fluid IL-8 was mildly but significantly associated with clinical severity independently of the questionnaire used. Partial correlation coefficients (PCC) and their corresponding 95% confidence intervals (CI) for the different severity scales were as follows: 0.291 (0.108 to 0.455) for Lequesne; − 0.216 (− 0.389 to − 0.029) for KOOS pain; − 0.221 (− 0.393 to − 0.033) to KOOS symptoms; and − 0.205 (− 0.380 to − 0.017) for KOOS function . When adjusted for confounders known to influence clinical severity as age, disease duration, KL and BMI, synovial fluid IL-8 remained essentially unaltered and statistically significant for Lequesne (PCC = 0.237; CI 0.045 to 0.411), KOOS pain (PCC = − 0.201; CI − 0.380 to − 0.008) and KOOS symptoms (PCC = − 0.209; CI − 0.387 to − 0.017). KOOS function also preserved the magnitude of it correlation with IL-8, although it did not retained statistical significance (PCC = − 0.185; CI − 0.365 to 0.009). None of these associations were observed for IL-8 in plasma (Table 2).

**Table 2:** Synovial fluid and serum IL-8 associations with clinical severity, inflammatory markers and adipokines in synovial fluid and blood.

	Synovial fluid IL-8^a^				Serum IL-8^b^			
	Univariate		Adjusted^c^		Univariate		Adjusted^c^	
	PCC [95% CI]	*p* value	PCC [95% CI]	*p* value	PCC [95% CI]	*p* value	PCC [95% CI]	*p* value
**Clinical severity**								
Lequesne	**0.291 [0.108, 0.455]**	**0.002**	**0.237 [0.045, 0.411]**	**0.016**	0.095 [− 0.093, 0.277]	0.320	0.101 [− 0.091, 0.287]	0.301
KOOS pain	**− 0.216 [− 0.389, − 0.029]**	**0.024**	**− 0.201 [− 0.380, − 0.008]**	**0.041**	− 0.028 [− 0.213, 0.160]	0.774	− 0.029 [− 0.219, 0.162]	0.764
KOOS symptoms	**− 0.221 [− 0.393, − 0.033]**	**0.021**	**− 0.209 [− 0.387, − 0.017]**	**0.034**	− 0.076 [− 0.258, 0.112]	0.431	− 0.071 [− 0.258, 0.121]	0.469
KOOS function	**− 0.205 [− 0.380, − 0.017]**	**0.032**	− 0.185 [− 0.365, 0.009]	0.062	− 0.059 [− 0.243, 0.128]	0.536	− 0.059 [− 0.247, 0.133]	0.548
**Inflammatory markers**								
TNF-α	**0.332 [0.143, 0.498]**	**< 0.001**	**0.334 [0.140, 0.503]**	**0.001**	0.082 [− 0.106, 0.265]	0.390	0.094 [− 0.099, 0.279]	0.340
IL-6	**0.455 [0.291, 0.594]**	**< 0.001**	**0.461 [0.293, 0.602]**	**< 0.001**	− 0.064 [− 0.248, 0.123]	0.502	− 0.034 [− 0.223, 0.158]	0.729
**Adipokines**								
Leptin	0.076 [− 0.119, 0.266]	0.445	0.006 [− 0.193, 0.204]	0.955	− 0.058 [− 0.241, 0.130]	0.547	− 0.036 [− 0.225, 0.156]	0.718
Adiponectin	0.095 [− 0.095, 0.279]	0.326	0.124 [− 0.071, 0.310]	0.211	0.068 [− 0.120, 0.251]	0.481	0.044 [− 0.148, 0.233]	0.654
Resistin	**0.201 [0.012, 0.376]**	**0.037**	0.182 [− 0.012, 0.362]	0.066	0.010 [− 0.176, 0.196]	0.914	0.024 [− 0.168, 0.214]	0.809
Visfatin	**0.256 [0.070, 0.424]**	**0.007**	**0.194 [0.000, 0.373]**	**0.049**	0.037 [− 0.151, 0.222]	0.702	0.037 [− 0.155, 0.226]	0.708
Osteopontin	**0.593 [0.455, 0.703]**	**< 0.001**	**0.575 [0.430, 0.692]**	**< 0.001**	0.059 [− 0.129, 0.242]	0.542	0.066 [− 0.126, 0.254]	0.502

^a^Synovial fluid associations between IL-8 with inflammatory markers and adipokines.

^b^Serum associations between IL-8 with inflammatory markers and adipokines.

*KOOS* Knee injury and Osteoarthritis Outcome Score, *SF* synovial fluid, *IL-8* interleukin-8, *IL-6* interleukin-6, *TNF-α* Tumor Necrosis Factor-alpha.

^c^Effects adjusted by measurement batch and by potential confounders: age, OA evolution time, KL grade and BMI. *KL* Kellgren–Lawrence scale, *BMI* Body Mass Index. Statistical significant associations are in bold type. *PCC* partial correlation coefficient. *95% CI* interval at 95% confidence.

Statistically significant results are highlighted in bold.

### Associations between IL8 and inflammatory markers

Moderate associations in synovial fluid samples between IL-8 levels and inflammatory markers were also observed, which retained statistical significance after control for potential confounders (age, disease duration, KL and BMI) without relevant changes in the magnitude of the associations. PCC estimations for these associations were 0.334 (CI 0.140 to 0.503) and 0.461 (CI 0.293 to 0.602) for TNF and IL-6, respectively. No association was detected in blood between IL-8 and any of these inflammatory factors (Table 2).

### Associations between IL8 and adipokines

When correlation with adipokines was evaluated, mild to moderate associations were observed in synovial fluid between IL-8 and resistin (PCC = 0.201; CI 0.012 to 0.376), visfatin (PCC = 0.256; CI 0.070 to 0.424) and osteopontin (PCC = 0.593; CI 0.455 to 0.703). Again, the magnitude of these correlations did not suffer important changes after controlling by potential confounders (age, disease duration, KL and BMI) for osteopontin (PCC = 0.575; CI 0.430 to 0.692) and visfatin (PCC = 0.194; CI 0.000 to 0.373), although statistical significance was not kept in the case of resistin (PCC = 0.182; CI − 0.012 to 0.362). No additional associations between plasma IL-8 and any of the plasma adipokines were observed (Table 2).

## Discussion

This study showed a weak association between synovial fluid IL-8 with clinical severity and local synovial fluid inflammatory markers, both with cytokines and adipokines. On the other hand, plasma IL-8 was not related to clinical severity nor blood cytokines or adipokines.

Previous studies found high levels of IL-8, both in synovial fluid and blood, in KOA patients compared to controls^10^. In contrast to our study, no clinical evaluation or inflammatory markers measurement were conducted in those works, and all KOA patients were analyzed during knee replacement surgery. Furthermore, the control group underwent diagnostic or therapeutic arthroscopy procedures for a knee injury, so, as usually in control groups compared to the OA sample, age-related differences exist. Ruan et al. found an association between plasma IL-8 with clinical (measured by WOMAC) and radiographic severity^11^, but as opposed to our study, men and women were included in this work, patients had no joint effusion and, consequently, synovial fluid was not analyzed. A recent study performed in synovial fluid found an association between IL-8 and KL stage, but it involved exclusively end-stage KOA patients who underwent prosthetic surgery with 45% of subjects evaluated in KL stage 4 and 40% in KL 3. In addition, no clinical evaluation was performed in the participants in this study^12^. Thus, to our knowledge, our study is the first to find that synovial fluid IL-8 is associated with clinical severity in knee OA patients.

Previous studies have associated resistin and ostepontin with IL-8 presence in chondrocytes and white adipose tissue in cultures from knee OA patients^13, 14^. The impact of IL8 on chondrocytes degradation could be related to OA progression and could point to new therapeutic approaches^15^. The association found in this work between IL-8 and clinical severity in knee OA together with its link with different inflammatory factors and adipokines might suggest that IL-8 could be among the last effectors in the inflammatory cascade of knee OA, as it is associated with a great array of molecules related to knee OA clinical severity.

A high level of IL-8 has been found in the serum and synovial fluid of patients with OA^16^. Different actions of IL8 could explain its involvement in OA, such as neutrophil chemotaxis, activation of leukocytes and migration to the joint or a direct effect on chondrocyte hypertrophy and differentiation or increased matrix metalloproteinase release^17–19^, as well as the induction of angiogenic changes related to chronic inflammation^20^. Within the joint, IL-8 is expressed by macrophages^18^, OA chondrocytes^19^, fibroblast-like synoviocytes^21^, and infrapatellar fat tissue^22^.

Our study has found an association for IL-8 and clinical severity and inflammatory markers in synovial fluid but not in plasma, which appears to support the hypothesis that changes related to OA physiopathology are more prominent in the joint, and synovial fluid should be the preferred biological fluid to be assessed. These results could indicate that, in this group of women with knee OA with join effusion, local inflammation is more relevant to clinical severity than systemic inflammation.

The main limitation of this study is related to its cross-sectional design, so that conclusions should be interpreted in terms of associations and we should be cautious about extrapolating causal relations. Another limitation is that as all patients were referred to our Rheumatology Unit for specialist care a selection bias towards more severe disease could exist. The lack of a control group did not allow us to confirm that interleukin-8 was specifically related to knee osteoarthritis symptoms and would be associated with clinical severity in other joint conditions associated with effusion. Additionally, these observations might be confirmed in a men cohort and were only associated in joint effusion related osteoarthritis women.

A strength of this work is the homogeneity of the patients in this study as only women with significant symptomatic knee OA and joint effusion, with predominantly low-to-moderate radiographic stage and moderate to high clinical severity were included. This homogeneity allows our results to be applied to a well-defined phenotype and increases the statistical power to detect moderate magnitude associations.

In conclusion, synovial fluid IL-8 was related to clinical severity in knee OA patients. The associations between IL-8 and several inflammatory markers and adipokines in synovial fluid, but no in blood, reinforces the hypothesis that local and not systemic inflammation is more relevant for clinical severity in these patients. Replication is warranted, especially in other groups of patients, such as men and different knee OA stages.

## Methods

### Study design and subjects

Patients systematically included in this cross-sectional study belong to a primary KOA cohort previously reported^23^. We studied 115 women aged 51–83 with symptomatic primary KOA according to ACR criteria and who showed significant joint effusion on physical examination and confirmed by ultrasound (≥ 4 mm on midline supra-patellar line). Included patients had pain intensity ≥ 4 on a 10-cm visual analogical scale despite the use of prescribed analgesic drugs for at least three months and had persisting knee effusion or documented effusion in several consultations. Patients with a history of trauma, meniscal injury, inflammatory rheumatic or septic arthritis, previous knee surgery or patients with any other secondary OA were excluded, as well as those any condition potentially influencing pain perception^4, 23, 24^. Patients who had received systemic glucocorticoids over the last six months or intra-articular glucocorticoid in the last three months or hyaluronic acid injection in the last 6 months were also excluded. Patients were recruited from October 2013 to June 2016. As there are gender-specific differences related to clinical severity measurement of OA and inflammatory markers or adipokines levels^25–27^ only women were included to homogenize the study sample. This study was approved by the Local Ethical Committee at the Hospital Universitari Parc Taulí, Sabadell (2013/591). All patients included were verbally informed and signed informed consent and all methods were performed in accordance with the relevant guidelines and regulations.

### Assessments

The following variables were collected: age, KOA disease duration and body mass index (BMI, kg/m^2^). Fasting blood analyses were carried out to assess serum inflammatory markers and adipokines. Joint aspiration was performed during the visit in fasting conditions and at the same time of day for proper evaluation of synovial adipokines. A minimum of 2 mL of aspired synovial fluid was required to include the patient in the study, and the mean and median across the cohort were 13.5 and 9 mL respectively. Non-inflammatory synovial fluid (cell count < 2000 cells) and absence of microcrystals were confirmed. Serum and synovial samples were stored at − 80 °C. Two validated scores (Lequesne index and Knee injury and Osteoarthritis Outcome Score (KOOS) were used to evaluate clinical severity. Subjects participating in this study complete only three out of five KOOS domains, specifically pain, symptoms, and function subscales. Low KOOS scores indicate a worse clinical severity in all domains. Radiographic severity was assessed according to the Kellgren–Lawrence scale (KL) with an antero-posterior knee X-ray examination in standing position performed over the last eighteen months. X-ray were evaluated independently by two rheumatologists (JC, CO).

As previously described, three inflammatory markers (IL-8, TNF-α and IL-6) and five adipokines (leptin, adiponectin, resistin, visfatin and osteopontin) were analyzed by ELISA following manufacturer recommendations for serum and synovial fluid dilutions. IL-6 and TNF-α were analyzed by Luminex HCYTOMAG-60 K-03 (Merck Millipore). Sensibility: IL-6: 0.9 pg/mL, TNF-alpha: 0.7 pg/mL, detection range: 3.2–2000 pg/L, Coef. intra-assay: IL-6: 2%, TNF-alfa: 2.6%, Coef. inter-assay: IL-6: 18.3%, TNF-alpha: 13%. Luminex IL8 Human Procartaplex simplex Kit (ThermoFisher Scientific) was used for analyzing IL8. The sensitivity of the assay tested on plasma is 1.2 pg/mL. Its intra-assay and inter-assay CV are respectively 8.5% and 4.6%. Adipokines were analyzed with Human Leptin ELISA Kit (Biocompare, California, USA). Dilution 1/100. Sensibility: < 8 pg/mL, detection rang: 62.5–10,000 pg/L, Intra-assay: < 6.3%, Inter-assay: < 7.2%. Adiponectin ELISA kit (eBioscience, California, USA). Dilution 1/1000. Sensibility: 0.01 ng/mL, detection rang: 0.78–50 mg/L, Coef. intra-assay: 4.2%, Coef. inter-assay: 3.1%. Human Resistin ELISA Kit (Raybiotech, GA, USA). Dilution 1/100. Sensibility: 1.4 pg/mL, detection rang: 1.4–400 pg/mL, Coef. intra-assay: < 10%, Coef. inter-assay: < 12%. Osteopontin ELISA kit (eBioscience, California, USA). Dilution 1/100. Sensibility: 0.26 ng/mL, detection rang: 0.47–30 mg/L, Coef. intra-assay: 6.7%, Coef. inter-assay: 6.1%. Visfatin ELISA kit (Phoenix Pharmaceuticals, California, USA). Dilution: none. Sensibility: 2.21 ng/mL, detection rang: 0.1–1000 ng/mL, Coef. intra-assay: < 10%, Coef. inter-assay: < 15%. Because of technical reasons inherent to ELISA (configuration of plates used), these markers could not be assessed at the same time for all patients, and non-negligible effects associated to time of measurement were detected^4, 24^. For this reason and in order to avoid biases in our estimations due to these effects that are technical in nature, the round of measurement was considered as an adjustment factor in all the statistical analyses performed in this study^4, 23, 24^. Although this correction allows reliable estimations of association for synovial and plasma measurements, it does not enable inference of either their real range of variability or meaningful cutoff values that can be extrapolated to external data, purposes for which a specific and specialized calibration study would be required^23, 24^.

### Statistical methods

Clinical and laboratory data were described using non-parametric methods. Medians and interquartile ranges were applied to continuous measures, whereas absolute and relative frequencies were used for categorical variables. For laboratory measures, differences in means due to measurement rounds were corrected previously to descriptive calculations. Association analyses were carried out by fitting linear models in which IL-8 was included as outcome, and inflammatory factors and adipokines were transformed suitably to meet the assumptions of the models. As mentioned above, all the models included round of measurement as covariate in order to account for the associated technical variability in the analyses (see “Assessments” section). As in previously published studies, associations were also assessed after statistical control by age, KOA symptom duration, radiographic stage evaluated by KL and BMI, which were included as explanatory variables in the models for this purpose. Partial correlation coefficients (PCC) and adjusted group means derived from the models were used to measure the magnitude of the effects for continuous and categorical variables, respectively^4, 23^.

A 5% was set as threshold for statistical significance. All statistical analyses were conducted using R.

### Ethics approval and consent to participate

Ethical approval was obtained from the Institutional Review-Board of the Parc Taulí University Hospital (Decision Number 2013/591). Participants signed informed consents.
